# The epidemiology of childhood brain injury in the state of Selangor and Federal Territory of Kuala Lumpur, Malaysia

**DOI:** 10.1186/s12887-016-0590-1

**Published:** 2016-04-27

**Authors:** Ee Lin Tay, Shaun Wen Huey Lee, Sabariah Faizah Jamaluddin, Cai Lian Tam, Chee Piau Wong

**Affiliations:** Tan Sri Jeffrey Cheah School of Medicine and Health Sciences, Monash University Malaysia Campus, Petaling Jaya, Malaysia; School of Pharmacy, Monash University Malaysia Campus, Petaling Jaya, Malaysia; Emergency and Trauma Department, Sungai Buloh Hospital, Petaling Jaya, Malaysia

**Keywords:** Traumatic brain injury, Incidence, Road traffic accident, Children

## Abstract

**Background:**

There are limited studies describing the epidemiology of childhood brain injury, especially in developing countries. This study analyses data from the Malaysian National Trauma Database (NTrD) registry to estimate the incidence of childhood brain injury among various demographic groups within the state of Selangor and Federal Territory of Kuala Lumpur.

**Methods:**

This study analysed all traumatic brain injury cases for children ages 0–19 included in the 2010 NTrD report.

**Results:**

A total of 5,836 paediatric patients were admitted to emergency departments (ED) of reporting hospitals for trauma. Of these, 742 patients (12.7 %) suffered from brain injuries. Among those with brain injuries, the mortality rate was 11.9 and 71.2 % were aged between 15 and 19. Traffic accidents were the most common mode of injury (95.4 %). Out of the total for traffic accidents, 80.2 % of brain injuries were incurred in motorcycle accidents. Severity of injury was higher among males and patients who were transferred or referred to the reporting centres from other clinics. Glasgow Coma Scale (GCS) total score and type of admission were found to be statistically significant, *χ*^2^ (5, *N* = 178) = 66.53, *p* < 0.001, in predicting patient outcomes. According to this analysis, the overall rate of childhood brain injury for this one year period was 32 per 100,000 children while the incidence of significant (moderate to severe) brain injury was approximately 8 per 100,000 children.

**Conclusions:**

This study provides an overview of traumatic brain injury rates among children within the most populous region of Malaysia. Most brain injuries occurred among older male children, with traffic, specifically motorcycle-related, accidents being the main mode of injury. These findings point to risk factors that could be targeted for future injury prevention programs.

## Background

Traumatic brain injury (TBI) is a major public health concern which contributes significantly to mortality and morbidity among youth [1, 2]. Previously published studies conducted in other countries such as in the United States, Australia and New Zealand have estimated the rate of childhood brain injury to range from 75 to 1,373 per 100,000 among children aged below 15 years old [3–6]. It is difficult, however, to accurately assess true incidence rates as these studies varied according to case ascertainment and inclusion criteria. For example, the two population based studies [4, 5] included cases presented to emergency departments (ED), hospital admissions and deaths. By contrast, the highest rate reported (1,373 per 100,000 children) [6] resulted from a longitudinal study of a single birth cohort, and included ED cases, hospital admissions and deaths, as well as cases presented to general practitioners (GP).

Data on incidence of TBI in South East Asian Nations (ASEAN) and other developing countries are not readily available. In Malaysia, a hospital-based study by Rohana and colleagues [7] estimated that 4.75 % of all paediatric cases admitted to the emergency department were related to TBI. This study was conducted more than a decade ago and, to the authors’ knowledge, there are no other published studies elating to childhood brain injury in Malaysia.

In this report the authors analysed the NTrD data on childhood brain injury in Malaysia in 2010. Specifically, the authors analysed in detail the incidence of childhood brain injury in the states of Selangor and Federal Territory of Kuala Lumpur, which fall within the same geographical region of Peninsular Malaysia, and together comprise about 20 % of the total population of the country [8].

## Method

### Data source

This was a cross-sectional, retrospective study analysing data from the National Trauma Database (NTrD). This database recorded information related to trauma patients in Malaysia from 2006 to 2010. Thirteen hospitals from various states in Malaysia participated as reporting centres for the registry. All tertiary referral hospitals (*N* = 6) operated by the Ministry of Health within the states of Selangor and Kuala Lumpur were reporting centres for this registry, and data from all (*N* = 6) these reporting centres was retrieved for analysis. Two tertiary academic hospitals within this region did not participate. A standardised form was used to collect data from each hospital [9]. Information collected included: a) patient’s demographic and clinical characteristics, b) admission details, c) injury related details such as mode and mechanism of injury, place of injury, and injury intent, d) diagnosis and operative procedures, as well as, e) patient outcomes. The Malaysian Institute of Road Safety Research (MIROS) oversaw data collection within participating hospitals for 2010. The MIROS database included all cases relating to road traffic accidents (RTA) while NTrD recorded major trauma cases. This ensured a comprehensive and fairly complete data collection, hence the data for 2010 was used for this analysis. Trauma patients aged between 0 and 19 years old and patients with head or brain injuries were included in the analysis.

### Measures

The NTrD contains several trauma scales which were used to classify the severity of patients’ injury. These include the Glasgow Coma Scale (GCS) score that measures one’s consciousness level [10], ranging from 3 to 15, with score of 13–15, 9 to 12 and 3 to 8 indicating mild, moderate and severe injury respectively. The other scale used was the Injury Severity Score (ISS) [11] which is an overall injury score derived from the Abbreviated Injury Scale (AIS). ISS scores range from 0 to 75, with higher scores indicating more severe injuries. GCS and ISS scores were assessed and documented by emergency department (ED) physicians on ED admission. Patients’ outcomes (alive or dead), discharge disposition and length of stay (LOS) were used as key predictors of morbidity and mortality.

### Statistical analyses

The age up to 19 was used to define the paediatric population in this analysis to align with the Malaysia intercensal population estimates denominator data [8] to facilitate calculation. Independent t-tests were performed to compare the severity of brain injuries (GCS) between gender, types of admission and for patients whom did or did not wear protective gear, especially helmets. Logistic regression was used to assess various prognostic factors on patient outcomes (alive or dead). IBM SPSS (version 20) was used for statistical analysis.

## Results

### Epidemiological data

From January 1, 2010 to December 31, 2010, a total of 5,836 patients aged 0–19 years old were admitted to ED of the six reporting hospitals with trauma. Of all trauma patients, 12.7 % (742) presented with brain injuries. Of brain injury patients, 75.1, 4.4 and 20.5 % had mild, moderate and severe brain injuries respectively. The median age of these patients was 17 (range from 0 to 19 years old). Males were 4 times more likely than females to suffer from TBI across all age groups. The highest rate of TBI admissions was observed in adolescent aged between 15 and 19 years old, who comprised 72.0 % (*N* = 534) of total admissions (Table 1).

**Table 1:** Baseline demographic and clinical characteristics of the study cohort (*N* = 742)

Variable		Number (%)
Gender	Male	594 (80.0)
	Female	148 (20.0)
Nationality	Malaysian	722 (97.3)
	Non-Malaysian	20 (2.7)
Age (years)	0–4	45 (6.1)
	5–9	30 (4.0)
	10–14	133 (17.9)
	15–19	534 (72.0)
GCS	Mild (13–15)	557 (75.1)
	Moderate (9–12)	33 (4.4)
	Severe (3–8)	152 (20.5)
ISS, Mean (SD)		6.74 (8.36)
Type of admission	Direct admission	551 (74.3)
	Transferred/ referred	191 (25.7)
Outcome^a^	Alive	652 (88.1)
	Death	88 (11.9)

^a^2 missing values

*GCS* Glasgow coma scale, *ISS* Injury severity score

About three quarters of the cases recorded (*n* = 551, 74.1 %) were direct admissions to the reporting hospitals while 191 (25.7 %) cases were referrals or transfers from other hospitals. The median duration of admission was 1 day (1 to 160 days) with an overall total of 3,200 hospital bed days accrued by the 742 patients. Seventy seven (*n* = 77, 10.4 %) patients were admitted to the intensive care unit (ICU). These patients spent a median of 4 days (1 to 105 days) and a total of 568 bed days in the ICU. Most of these patients were alive at discharge, 11.9 % (*n* = 88) of the patients died (Table 1). The mode of brain injuries is illustrated in Table 2. The most frequent mode of injury was RTA, which accounted for almost 95.4 % of all injuries recorded.

**Table 2:** Mode of brain injury

Mode of injury	Age Group					
	0–4	5–9	10–14	15–19	Total	Percentage (%)
Road traffic accident	27	27	132	522	708	95.4
Falls	15	3	1	2	21	2.8
Industrial accident	0	0	0	3	3	0.4
Others	1	0	0	6	7	1.0
Unknown	2	0	0	1	3	0.4
Total	45	30	133	534	742	100.0

### Injury variables

**Fig. 1:**
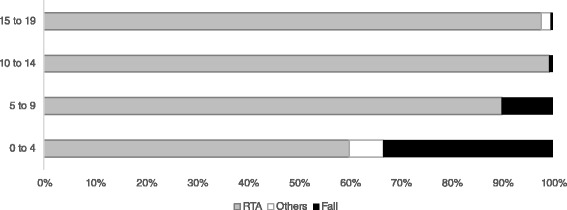
An inverse trend of distribution of road traffic accident (RTA) and fall as mode of injury according to age is shown. The percentage of RTA was higher among children aged 10 years and above while the percentage of fall was higher among children below 9 years old

RTA occurred more frequently among patients aged 10 years and above (*n* = 654, 92.4 %) while falling as mode of injury was more frequent among children below 9 years old (*n* = 18, 85.7 %; Fig. 1). RTA involved mainly motorcycle accidents (*n* = 595, 80.2 %). Data on the use of helmet among RTA survivors with TBI was available in 441 patients, who were either cyclists or motorcycle riders. Helmets were worn in 67.3 % (*n* = 297) of cases, while 32.7 % (*n* = 144) of riders were not wearing helmets at the time of the accidents. The use of helmets was found to reduce the severity level (GCS) of injuries (*Mean* =14.35, *SD* = 2.357) in comparison to those who did not wear helmets (*M* =13.51, *SD* = 3.547), t (408) = 2.876, *p* = 0.004.

There were significant differences in injury severity based on gender and type of admission. Higher severity was observed in males based on GCS (*M* =12.30, *SD =* 4.536) compared to females (*M* = 13.59, *SD =* 3.610), *t* (738) = −3.203, *p* = 0.001). Patients who were transferred or referred to the reporting centres (*M* = 10.99, *SD =* 4.971) had more severe brain / head injuries than patients who were admitted directly (*M* = 13.11, *SD =* 4.039), *t* (738) = 5.860, *p* < 0.001 (Table 3). GCS scores also correlated negatively with the length of hospital stay (LOS) (*r* = −0.369; *p <*0.001) but not length of ICU stay (*r* = −0.083; *p >* 0.05). As expected, patients with higher GCS scores had lower ISS scores (*r* = −0.556; *p <*0.001).

**Table 3:** Summary of variables and difference in injury severity

Variable	Mean (SD)	Injury Severity (GCS)
Gender		
- Male	12.30 (4.536)	
- Female	13.59 (3.610)	*P* = 0.001
Type of admission		
- Transferred/ Referred	10.99 (4.971)	
- Direct	13.11 (4.039)	*p* < 0.001
Use of helmet		
- Yes	13.58 (3.631)	
- No	13.07 (4.003)	*P* > 0.05

### Predictors of survival

This analysis used a logistic regression model containing five independent variables (gender, age, type of admission, GCS total score, and total ISS) to predict survival. The original model containing all five factors was statistically significant, *χ*^2^ (5, *n* = 178) = 66.53, *p* < 0.001, in predicting patient outcomes. However, as shown in Table 4, when accounting for the inter-relationships among the predictors, only two factors remain statistically significant (GCS total and type of admission). The strongest predictor of patient outcome was type of admission, with odds ratio (OR) of 5.63 (95 % CI 2.61 to 12.15), whereby patients who were admitted directly were much more likely to survive. Patients with higher GCS scores were more likely to survive (OR 1.53, 95 % CI 1.30 to 1.79).

**Table 4:** Logistic regression predicting the patients’ outcomes

	B	S.E.	Wald	df	*p*-value	Odds ratio	95 % CI	
							Lower	Upper
Gender	.999	.584	2.924	1	.087	2.715	.864	8.528
Age	.005	.042	.013	1	.910	1.005	.926	1.090
GCS	.422	.080	27.511	1	.000	1.525	1.302	1.785
Adm	1.727	.393	19.338	1	.000	5.626	2.605	12.149
ISS	−.018	.018	1.022	1	.312	.982	.949	1.017

GCS, GCS total score; Adm, types of admission; ISS, total ISS. Cox and Snell R^2^ = 0.312. Nagelkerke R^2^ = 0.417. Correct classification = 79.8 %

### Incidence

The estimated population aged 19 years old and below was 2,274 678 in the 2010 population census [8]. Consequently, the calculated incidence of childhood brain injury based on the 2010 NTrD was approximately 32 per 100,000 children. Moderate to severe brain injury (significant), which usually leads to death or significant sequelae was approximately 8 per 100,000 children.

## Discussion

This analysis provides an insight into the epidemiology of childhood brain injuries in Malaysia. Brain injury was found to be much more frequent among children from 15 to 19 years old (72 %) as well as among males (80 %). The most common mode of injury was road traffic accidents (RTA, 95.4 %). More specifically, motorcycle accidents accounted for about 80 % of traffic accident-related TBI. This study provides the first comprehensive overview of childhood brain injury within the most populous region of Malaysia in the last 5 years. The standardized form of data collection across participating hospitals strengthened the reliability and accuracy of the data analysed.

Male patients were more likely than females to have been injured in RTA across all age groups.

Males in this study were 4 times more likely to suffer from TBI than their female counterparts and the vast majority of them were RTA victims. This trend is similar that seen in most previous studies where the ratio of male to female TBI tends to be around 2:1 [3, 4, 7, 12, 13]. This is likely to be due to higher risk-taking behaviour among males [14]. This is similar to a previous study which shows that Malaysian male drivers are three times more likely than female drivers to be involved in RTA [15].

In the current study, RTA was, by far, the leading mode of brain injury, accounting for almost 95.4 % of all cases reported, out of which over half were adolescents aged between 17 and 19 years. However, this should be interpret with caution as the data could have skewed toward RTA as the main cause given the involvement of MIROS throughout the data collection period. Nevertheless, this is still consistent with a previous study [16] showing that Malaysia has the highest rate of RTA fatalities among ASEAN countries. More than half of these fatalities are motorcyclists aged 16 to 20 years old [16]. Pedestrians, motorcyclists and cyclists are the usual casualties in RTA [17]. Such numbers clearly point to a need for improving traffic safety in Malaysia.

Falls were the most common mode of traumatic brain injury among children aged 9 and below. However, the rate of falls was lower (18 out of 75 cases, 24 %) in comparison to Rohana’s [7] study which reported 63 % of fall related injuries. Although the cause of falls was not recorded in the NTrD, lack of adult supervision was reported as the main cause of accidents by Rohana. This decline may be attributed to the enforcements of various child maltreatment (neglect and abuse) prevention programmes [18], the enforcement of Child Act 2001 [19] and increased awareness among the public.

Our results are similar to previous studies that have indicated a bimodal age-related distribution of TBI: We observed an initial peak among younger children below 4 years old and another among older children over 15 years old [4, 6, 20–22]. These changes appear to relate to the mode of injuries as previously eluded.

The overall incidence of childhood brain injuries in this analysis was 32 per 100,000 children with the incidence of significant childhood brain injuries at 8 per 100,000 children. These results are much lower than other studies. For example, childhood brain injury ranges have been estimated at 280 per 100,000 children in United Kingdom and 842 per 100,000 children in the United States [3, 4]. These rates, however, cannot be compared directly due to different methodologies and inclusion criteria. Nevertheless, the incidence of significant brain injuries in our analysis was fairly similar to that conducted by B Mitra, P Cameron and W Butt [5] in Australia. Using similar inclusion criteria, they reported a TBI rate of 7 per 100,000 children per year. These vastly different results support Roozenbeek’s [17] call for greater standardization in epidemiological monitoring of TBI. The incidence found from this analysis should be a good approximation of the actual incidence. This is because most brain injuries in Malaysia usually present to hospitals ED and rarely treated by the general practitioners or at home. Whilst mild brain injuries may be seen in private hospitals, moderate to severe injuries are usually referred to (by private hospitals) the public hospitals in MOH, especially patients who have no insurance cover.

In this study, patients who were admitted directly to the reporting hospitals were 5 times more likely to survive than those who were transferred or referred from other hospitals or clinics. This is likely to be due to the fact that, most severe cases were usually brought to the reporting hospitals directly (which are tertiary hospital with appropriate expertise to care for these patients). The increase in mortality in the referred cases could also be due to the delay in managing these cases caused by the transfers. Primary admissions to reporting hospitals are more likely to include patients with milder injury and a much wider range of injuries. There other many possible contributing factors to this finding such as the adequacy of care of the primary hospital or the transport system. This is beyond the scope of this current analysis. Nevertheless, this result suggests that a more comprehensive evaluation of mortality rates across health care providers may be warranted. Such a project should include detailed evaluations of local trauma care and referral systems [5], as well as methods of regionalizing trauma care to ensure that patients promptly receive appropriate medical care [23]. Such regionalization could also improve organizational efficiency and allocation of resources to ensure better health care delivery.

There are several limitations in this study. As this research was retrospective in nature, the data evaluated may have included selection biases. The collaboration with MIROS in 2010 might have caused the data to skew towards RTA as the main mode of brain injury. The National Trauma Database (NTrD) registry only collected data from tertiary hospitals overseen by the Ministry of Health. Data from academic centres and private hospitals was not included in this registry. Consequently, the rates found in this analysis are an underestimation of the actual incidence of TBI. Nevertheless, as previous studies indicate that 70 % of TBI present to public hospitals [24], we can assume that the data used in this study represent approximately 70 % of total TBI cases in this region of Malaysia.

## Conclusions

To the authors’ knowledge, there has been no published research on the epidemiology of childhood brain injury in Malaysia for the past 17 years. Thus, this study provides an important update in this area. The incidence of childhood brain injury was 32 per 100,000 children. In general, our findings indicate that the incidence of childhood brain injury in Malaysia follows similar patterns to that seen in other countries. The findings regarding the incidence rates of significant childhood brain injury are similar to other studies that used similar methodology and inclusion criteria. The study also reinforced the need to continue the traffic safety awareness initiatives and programmes especially among motorcyclists. In addition standardized data recording as well as more extensive post-discharge follow-up and data collection will be useful to understanding patients’ recovery processes and enable the optimal provision of rehabilitation services.

### Ethics approval and consent to participate

Ethics approval was obtained from the National Medical Research and Ethics Committee (MREC) for the NTrD registry (NMRR 05-01-158). Public notice as a form of consent was approved by the MREC for all participants of the registry. These opt-out notices were placed at various treatment and waiting areas in the reporting centres. Permission was obtained from the registry committee to analyse the data for this study. Ethics exemption from Monash University Human Research Ethics Committee (MUHREC) has also been obtained (CF14/1869 – 2014000962).

### Availability of data and materials

The authors do not have the permission from NTrD registry to republish the raw data. In order to access the data, kindly request permission from the registry via this link (http://www.acrm.org.my/ntrd/).

## Abbreviations

AIS: abbreviated injury scale
ED: emergency department
GCS: glasgow coma scale
ICU: intensive care unit
ISS: injury severity score
LOS: length of hospital stay
MIROS: Malaysian institute of road safety research
NTrD: national trauma database
OR: odds ratio
RTA: road traffic accident
TBI: traumatic brain injury
